# A First-Principles Study on the Multiferroic Property of Two-Dimensional BaTiO_3_ (001) Ultrathin Film with Surface Ba Vacancy

**DOI:** 10.3390/nano9020269

**Published:** 2019-02-15

**Authors:** Haigen Gao, Zhenxing Yue, Yande Liu, Jun Hu, Xiong Li

**Affiliations:** 1 School of Mechatronics & Vehicle Engineering, East China Jiaotong University, Nanchang 330013, China; gaohg@tsinghua.edu.cn (H.G.); jun1_hu@163.com (J.H.); xiongv_li@163.com (X.L.); 2 State Key Laboratory of New Ceramics and Fine Processing, School of Materials Science and Engineering, Tsinghua University, Beijing 100084, China

**Keywords:** first-principles, two-dimensional BaTiO_3_ ultrathin film, surface Ba vacancy, magnetoelectric coupling

## Abstract

In this work, the multiferroic property of Ba-deficient BaTiO_3_ (001) ultrathin film is studied employing the first-principles approach. The BaTiO_3_ (001) ultrathin film is more energetically stable and behaves as a semiconductor relative to the (111) and (101) configurations, confirmed from the surface grand potential and electronic density of states. The electronic structures show that the O vacancy can switch the (001) film from a semi-conductor into a metal, while the Ba defect has a slight influence on the band gap, at a concentration of ~2.13%. In Ba-deficient (001) film, the spontaneous polarization pattern is changed and a spontaneous polarization parallel to the surface is observed. Furthermore, a magnetic moment is induced, and it is found to be originated from the O atoms in the supercell. Our results suggest that a strong magnetoelectric coupling occurs because the magnetic moment exhibits a 43.66% drop when the spontaneous polarization increases from 12.84 *µ*C/cm^2^ to 23.99 *µ*C/cm^2^ in the deficient BaTiO_3_ with *m* = 2 under the bi-axial compress stress field.

## 1. Introduction

Multiferroics are the materials possessing two or more ‘ferroic’ orders, such as ferroelectricity, ferromagnetism, ferroelasticity and ferrotoroidicity. Of them, the coupling between the ferroelectricity and magnetic order exhibits a great prospect to be applied in the novel spin-based or nonvolatile data-storage devices where the information is written electrically and read magnetically, and attracts many interests of research [1,2,3,4,5,6]. Besides that, it can also be employed in the multifunctional transducers, advanced memory, acoustic and microwave devices etc. [7,8,9]. The magnetoelectric (ME) effect results from the intrinsic interactions between lattice, charge, spin, and orbital degrees of freedom [10,11,12,13]. Although the single-phase multiferroic materials have been found, their ME couplings are generally weak [11,12,13,14,15,16,17]. For example, the investigations show that the ME coupling coefficient of BiFeO_3_ nanowire is ~2.2 × 10^−10^ s m^−1^ [18], and that of Cr_2_O_3_ is low to ~4.1 × 10^−12^ sm^−1^ [19]. Therefore, it is highly encouraged to discover new single-phase multiferroic materials with strong ME coupling. In practice, the mangetoelectrics can be created based on the strong single-phase ferromagnet/ferroelectricity or the composites of strong ferromagnetic and ferroelectric [20,21,22,23]. In addition, to replace and distort the crystal structure of strong ferromagnetic and ferroelectric compounds is also available, as well as the composites with high magnetoelectric coupling [24,25,26]. The substituted M-type hexaferrites are found to behave in the manners of deep semiconductors and exhibit good magnetic properties, such as the Al-, In- and Ga-doped compounds etc. [17,18,27,28,29]. Furthermore, the large spontaneous polarization and ME coupling at room temperature are also discovered in the M-type hexaferrites doped with the diamagnetic cations [30,31,32]. Trukhanov et al. reported that a high spontaneous polarization of 5.8 mC/m^2^ was observed in the Al-substituted barium hexaferrites, and the ME coupling coefficient was measured to be 0.032, which is nearly ten times higher than that of BiFeO_3_ [30,33]. However, with the development of industries, the downsizing of electronic devices is necessary to meet with the market demands. Consequently, the thin/ultrathin films are prepared to instead of the bulk materials as compositions in the devices with aid of the advanced atomic deposition technologies. When the thickness is thinned to the nanometer or several unit cells, the spontaneous polarization in the doped M-type hexaferrites would be depressed. The corresponding two-dimensional hexaferrites with high spontaneous polarization has been rarely reported. It can be illustrated that the applications of M-type hexaferrites as multiferroic materials in the nanodevices are unavailable at current. In recent decades, low-dimensional functional materials are under active explorations, due to their new and rich physical properties and the great potential prospect to be applied in nanoelectrionics [34,35,36,37,38]. For an instance, in the study of ferroelectric materials that attract extensive attentions as a major constitution of the current electronic devices, the Ca_2_Nb_3_O_10_^−^ and Sr_2_Nb_3_O_10_^−^ nanosheets were reported with a permittivity of 210–230 [34], matching that of bulk. It reveals that the low-dimensional dielectric ultrathin film or nanosheet can be used as the dielectric layer to be applied in the nanocapacitors. 

Since 2004, the ferroelectric domain has been observed in the ultrathin PbTiO_3_ film with the thickness from 1 to 4 unit cells prepared by Dillon et al., it stimulates much interest of research to give an insight into its screening mechanism of *depolarizing* field and ferroelectric property [39]. Shimada et al. found that the *depolarizing* filed is screened by the formation of in-plane spontaneous polarization which matches that of a bulk calculated using the first-principles methods [40]. Similar with the PbTiO_3_, the BaTiO_3_ is also one of the typical ferroelectric materials with perovskite structure (ABO_3_), which takes advantages of the high dielectric constant and low dielectric loss, and has no toxic elements, such as Pb element [41]. Furthermore, its spontaneous polarization can be controlled by the external stress; when the applied compressive strain achieves to −3.8%, the spontaneous polarization is enhanced to 42.7 *µ*C/cm^2^ [42], which is higher than the value of 26.0 *µ*C/cm^2^ in bulk [43]. Besides that, a magnetic moment can be induced in the BaTiO_3_ by the formation of O vacancy defects, however, it is confirmed to behave like a conductor [44]. One should also be cognizant of that the surface vacancy O, Ti or Ba is readily introduced in the preparation process for BaTiO_3_ ultrathin film. In principle, the ultrathin film with Ba or Ti vacancy is a Kohn-Sham insulator or semi-conductor, because it can be considered as an acceptor doping. It is interesting to explore whether the ferroelectricity and magnetism could coexist in the Ba or Ti deficient BaTiO_3_ ultrathin film.

## 2. Computational Method

In this work, the model of BaTiO_3_ ultrathin film with thickness *m* defined from one to three unit cells is constructed. Electronic structure, magnetic property and spontaneous polarization are studied using the first-principles plane-wave pseudopotential method, based on density functional theory (DFT) as implemented in the Vienna *ab* initio simulation package (VASP5.3) [45,46]. The projector augmented-wave pseudopotentials with generalized gradient approximation (GGA) of PW91 are used in the calculations [47]. In the study of the interaction between spontaneous polarization and magnetic moment, the PBEsol functional is also employed to perform as a comparison [48]. The semi-core Ba 5*s* and 5*p* and Ti 3*s*, 3*p*, 3*d* and 4*s* orbitals are treated as valence states. In the case of PBEsol, plane-wave energy cutoff is set as 520 eV, while the cutoff is fixed as 400 eV for PW91, and the residual forces are less than 0.01 eV/Å in the geometry optimization by using the conjugate gradient algorithm [49,50]. Simulation models contain three, five and seven layers along the *z*-axis, and the vacuum has a capacity of 4 unit cells of BaTiO_3_ above the top surface and below the bottom surface. The Brillouin zone integration is calculated using a k-point mesh of 5 × 5 × 3 for *m* = 1 and 2, and 1 × 5 × 1 for *m* = 3, automatically generated with the Monkhorst-Pack method [51]. For the spontaneous polarization calculation, the berry-phase and density functional perturbation theory (DPFT) methods as implemented VASP are used to estimate spontaneous polarization for bulk and ultrathin film [52,53,54].

## 3. Results and Discussion

**Figure 1:**
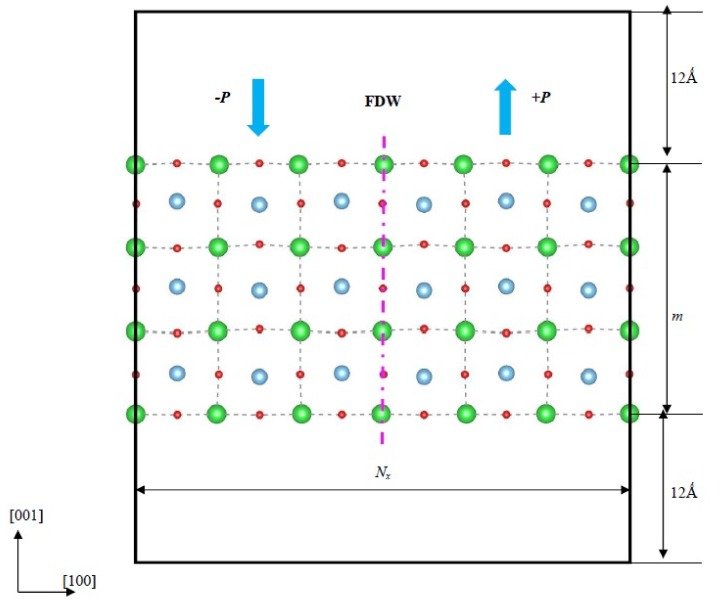
The simulation model of BaTiO_3_ (001) ultrathin film, the *N_x_* denotes the domain period, and the *m* is the total unit cell in the thickness and used to represent the thickness.

To search the most stable configuration that can be prepared in the experiment, two-dimensional BaTiO_3_ ultrathin films with the (001), (101) and (111) surfaces are studied using the first-principles method in this work. At first, the crystal structure of bulk BaTiO_3_ are optimized to set the appropriate simulation parameters, and the results are evaluated to be *a = b* = 3.979 Å/*c* = 4.064 Å and *a = b* = 3.987 Å/*c* = 4.067 Å, with the PBEsol and PW91 functional respectively, which are in good agreement with the theoretical value *a = b* = 3.980 Å/*c* = 4.076 Å and the experimental data *a = b* = 3.997 Å/c = 4.0314 Å [55,56]. Figure 1 is the simulation model of BaTiO_3_ (001) ultrathin film, where the +*P* and −*P* are used to represent the up and down spontaneous polarization perpendicular to the surface, the +*P* is the spontaneous polarization oriented out of the top surface, and −*P* is that orientated into the top surface. For an accurate simulation, the vacuum layer above and below the top and bottom surface is set as 12 Å.

In order to compare the stability of the films with different surfaces, the surface grand potential is employed. Different from the formation energy, the surface grand potential not only can determine the stability, but also can give the information of the practical surface environment condition, such as the chemical composition of termination. Hence, it is used to study the film’s stability and defined as follows [57]:

(1) $$E_{s}=(E_{slab}^{tot}-n_{i}\mu_{Ba}-n_{j}\mu_{Ti}-n_{k}\mu_{O})/A,$$

where *E_slab_^tot^* is the total energy of two-dimensional BaTiO_3_ film, *n_i_*, *n_j_*, *n_k_* and *µ_Ba_*, *µ_Ti_*, *µ_O_* are the total amounts and the chemical potentials of Ba, Ti and O atoms, respectively, and *A* is the surface area of ultrathin film. Here, bulk cubic Ba, hexagonal Ti and molecular oxygen are adopted to evaluate the chemical potentials of Ba, Ti, and O atoms.

**Figure 2:**
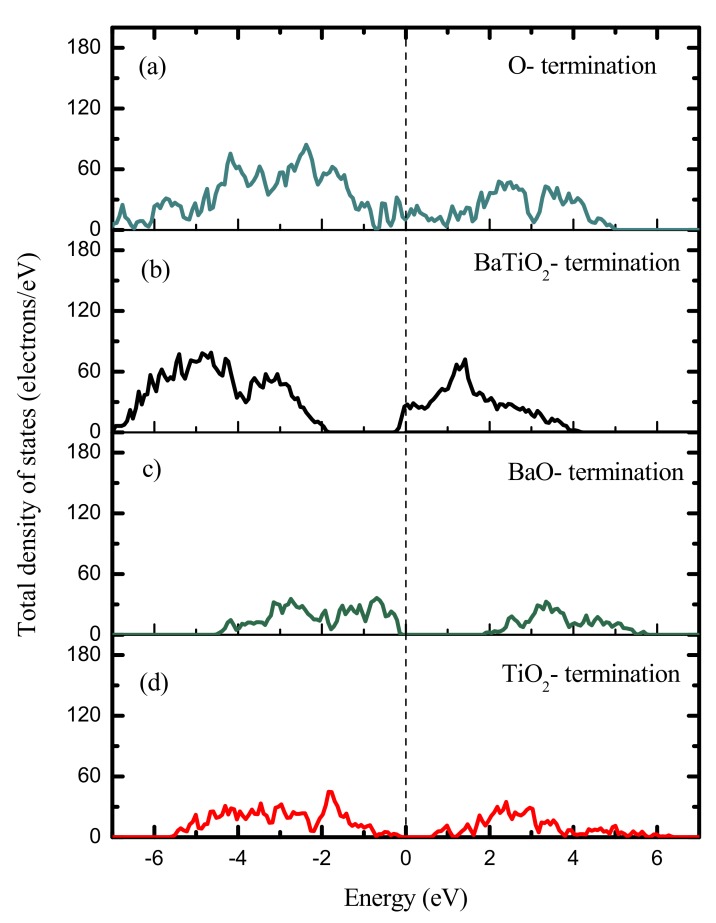
Total electronic densities of states of paraelectric BaTiO_3_ (101) films with thickness *m* = 6 and ferroelectric (001) ultrathin films with thickness *m* = 2, corresponding to different terminations. The (101) ultrathin films with (**a**) O- termination and (**b**) BaTiO_2_- termination, and the (001) ultrathin film with (**c**) BaO- termination and (**d**) TiO_2_- termination.

For simplicity, the paraelectric simulation model is built with the thickness *m* = 6 (*m* is the total number of BaTiO_3_ unit cells in the film thickness) and the dimension of 1 × 1 × 6 unit cells, where the two layers in the middle are fixed. And the (101) and (111) ultrathin films’ structures are given in the supplementary as Figures S1 and S2. That’s because it is found that the total energy differences between the BaTiO_3_ ultrathin films of the cubic and tetragonal crystal structures are slight. Table 1 is the corresponding calculated surface grand potentials, it shows that the (111) ultrathin film is the most energetically unstable because the potentials −41.62 *J/m*^2^ and −38.38 *J/m*^2^ of the BaO_3_- and Ti- terminations are the highest. While in the case of the (101) film, the data for the BaTiO- and O_2_- terminations are −75.52 *J/m*^2^ and −62.12 *J/m*^2^ respectively, which are the lowest. However, the electronic density of states (DOS) demonstrates that they behave as conductors, as shown in Figure 2a,b. Figure 2c,d are the DOS’s of the (001) ultrathin films with BaO- and TiO_2_- terminations, it shows that both of them are semiconductors with a ~2.00 eV and ~0.50 eV Kohn-Sham band gap, and in good agreement with that reported by Dionot et al. [24]. It is noted that the band gaps of the (001) films used to compare the stability (*m* = 6) are ~1.63 eV and ~0.43 eV, and that in Figure 2c,d are calculated from the (001) configurations with thickness *m* = 2. This means the existence of band gaps for the (001) ultrathin films persists with different thicknesses. Moreover, the (001) surface has been observed experimentally indicating the feasibility for the fabrication of the (001) ultrathin films [58]. For these reasons, we focus our study on the (001) ultrathin film. Table 1 shows that the surface grand potential of TiO_2_- termination is lower than that of BaO- termination, but their difference is only 1.44 *J/m*^2^. To further determine the stable structure, the total energy difference between the (001) ferroelectric polydomain and paraelectric phase as a function of the domain period is also studied, and it is predicted as follows [23]:

(2) $$\Delta E=\frac{E_{f}-E_{p}}{N_{x}},$$

where Δ*E* is the total energy difference between the ferroelectric polydomain relative to paraelectric phase, *E_f_* and *E_p_* are total energies of ferroelectric and paraelectric films. *N_x_* is the domain period of the ultrathin film, or the total number of BaTiO_3_ unit cells in the domain period in the direction [100], as shown in Figure 1.

**Figure 3:**
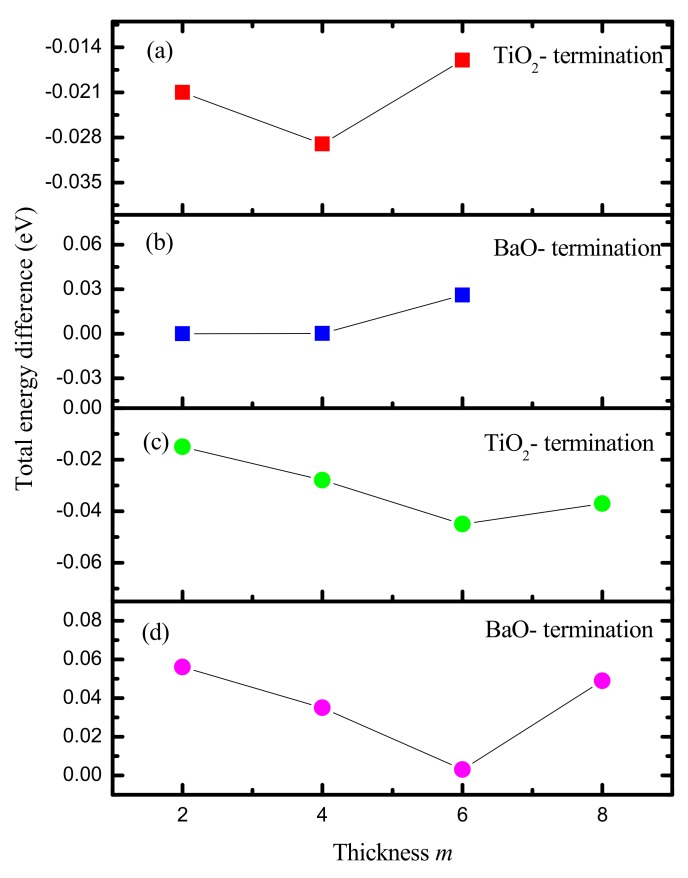
The total energy differences between polydomains and paraelectric phases of BaTiO_3_ (001) ultrathin films with different terminations and thicknesses *m*. (**a**), (**b**) *m* = 2, (**c**), (**d**) *m* = 3.

The total energy differences Δ*E* are plotted in Figure 3, it exhibits clearly that the ferroelectric structure is more stable than the paraelectric one in the case of TiO_2_- termination. And for the BaO- termination, the paraelectric phase is more stable. In addition, we also find that the total energy difference Δ*E* has the lowest positive and highest negative value for the BaO- and TiO_2_- termination of *N_x_* = 6 when the thickness *m* is 3, indicating that they are the most stable configurations with *m* = 3. In the same way, *N_x_* is determined to be 4 and 2 for the TiO_2_- and BaO- terminations in the case of thickness *m* = 2. In practice, both the paraelectric and ferroelectric structures can be considered stable since the the total energy differences Δ*E* and surface grand potentials *E_s_* are rather small observed from Figure 3 and Table 1. Taking the electronic structure into account, the configuration with BaO- termination is adopted to study the ME coupling because of its wide band gap of ~2.00 eV. In further, it is experimentally achievable by the molecular beam epitaxy method, due to the quite small or negligible stability difference between the TiO_2_- and BaO- terminations, especially in the case of *m* = 3, where the films are deposited at 5 × 10^−6^ torr of molecular oxygen at 750 ^o^C with a growth rate of 1 unit cell per 2 min, and are crystalline as deposited, then cooled down to 200 °C with a ramp rate of 30 °C /min in an oxygen environment of 5 × 10^−6^ torr [58].

**Figure 4:**
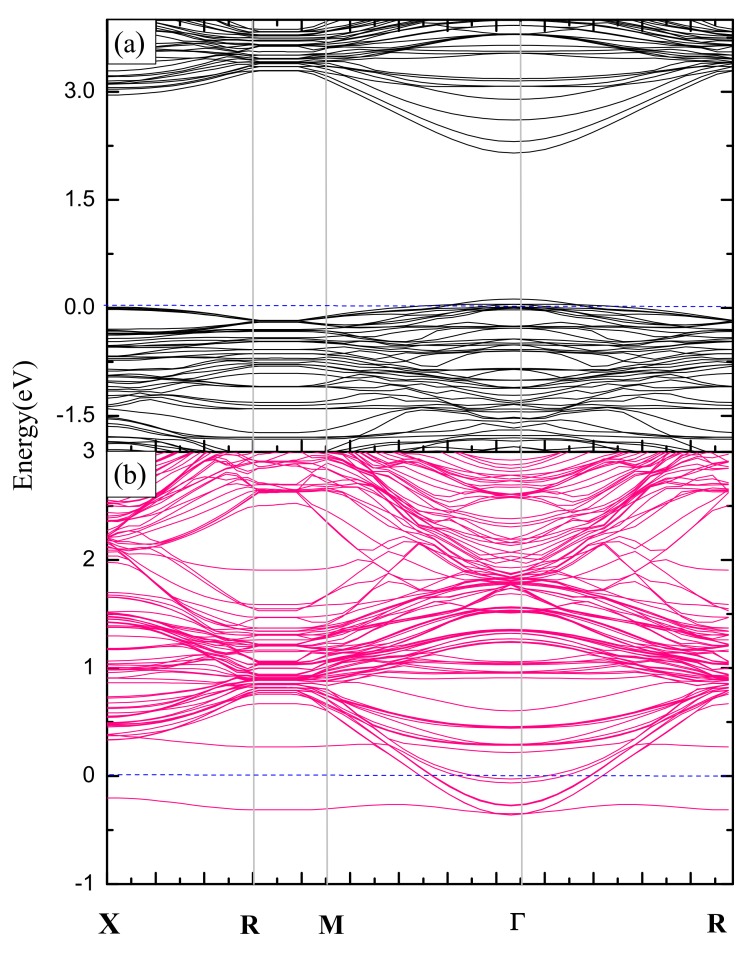
Band structures of BaTiO_3_ (001) ultrathin films with the surface Ba and O vacancy defects, where the thickness *m* is 2. (**a**) Ba vacancy, (**b**) O vacancy.

In the growth of ultrathin BaTiO_3_ film, the surface vacancy is unavoidably produced, such as the Ba and O vacancies, etc. It can be imaged that the ultrathin film will be an oxygen excess or deficit configuration, in which the states of 3d metals would be changed, as a result, the magnetic moment may be induced as the reports indicated [59,60]. There, the magnetic properties and ME couplings of the anion-deficient La_1-*x*_Ba*_x_*MnO_3-*x*/2_ and Co*_x_*Zn_1−*x*_Fe_2_O_4_ are greatly affected by the oxygen nonstoichiometry. Furthermore, these vacancies will also heavily influence the corresponding electronic structures owing to the small atomic scale in the thickness. Hereby, the formation of the Ba and O vacancy is studied to determine its stoichiometry, and the corresponding formation energy is calculated using the following Equation (3) [61]:

(3) $$E_{f}=E^{tot}(ref+V)-E^{tot}(ref)+\mu_{a}$$

where *E^tot^*(*ref + V*) is the total energy of the ultrathin film with a vacancy, *E^tot^*(*ref*) is the total energy of the film without defects, and *µ_a_* is the chemical potential of the atom removed to form a vacancy. Here, the ultrathin film with thickness *m* = 2 is taken as an example. Formation energies are calculated to be 7.58 eV and 5.13 eV, corresponding to surface Ba and O vacancies, respectively, which suggests that the O vacancy is more readily produced, leaving the ultrathin film with redundant Ba atoms. To gain an insight into the influence, of surface vacancy on the electronic structure, the band structure of (001) film with a surface Ba or O vacancy and thickness *m* = 2 is calculated, as shown in Figure 4a,b. It demonstrates that the (001) film with a ~2.13% concentration of O vacancy acts as a metal (the concentration is calculated from the number ratio of the vacancies vs the total atoms in the supercell), while that of Ba vacancy is still a semi-conductor, and the band gap is slightly higher than 2.00 eV. To obtain the Ba-deficient film, the stoichiometric ratio of Ba and O atom in preparation should be controlled to produce Ba vacancy.

**Figure 5:**
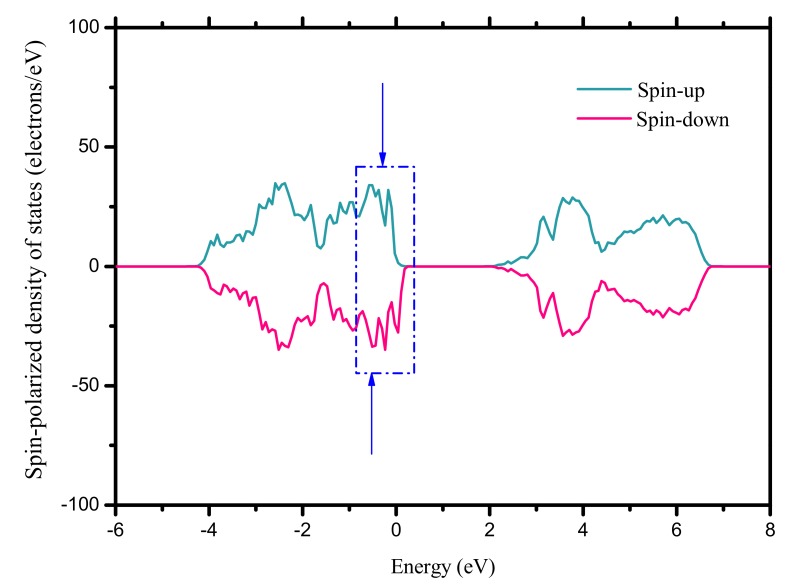
Spin-polarized density of states of BaTiO_3_ ultrathin film.

**Figure 6:**
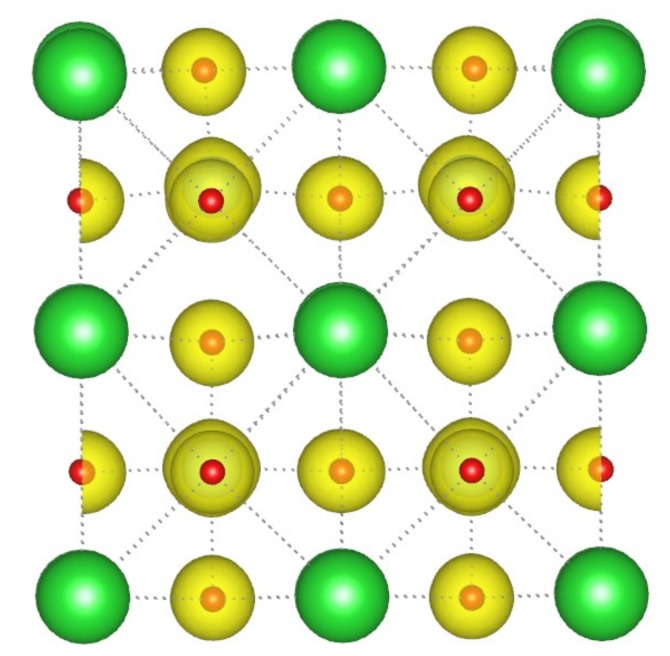
Spin-polarized charge density of BaTiO_3_ ultrathin film with thickness *m* = 2.

As discussed above, it can be readily imaged that the orbital hybridization pattern between the atoms is changed when the surface Ba vacancy is formed. Consequently, the magnetic properties of BaTiO_3_ ultrathin film could be different relative to the perfect configuration since it can be considered as an oxygen excess and hole doping. Notably, the spin-up and spin-down DOS’s do not match with each other for the film with thickness *m* = 2 as plotted in Figure 5. It suggests that a magnetic moment is introduced, and it is calculated to be 1.81*μ_B_* (0.23*μ_B_/unit cell*) in total at a doping concentration of ~2.13%. For the thickness *m* = 1, the total magnetic moment is determined to be 2.00*μ_B_* (0.50*μ_B_/unit-cell*) with a ~7.69% dopant concentration. For comparison, the total magnetic moment calculated using PBEsol functional is ~2.00*μ_B_* (0.50*μ_B_/unit-cell*) and 1.97*μ_B_* (0.25*μ_B_/unit-cell*) respectively for the configuration with *m* = 1 and 2, confirming the existence of magnetic polarization. It should be pointed out that the oxygen excess has no influence on the states of the Ti^4+^ and Ba^2+^ cations, but it can produce holes in the film and has an influence on the magnetization. To sake for its mechanism, the magnetic moment as a function of concentration of Ba vacancy for thickness *m* = 1 and 2 are studied. The results show that they are in a noncollinear relationship. To exclude the surface effect, the spin charge density distribution of ultrathin film with thickness *m* = 2 is plotted, as shown in Figure 6. It can be determined that the introduction of magnetic moment is mainly originated from the O atoms in the supercell. That’s to say, the oxygen excess is in controlling of the magnetic moment. Further, the calculation of magnetic moment on the ultrathin film with *m* = 3 is also done, where both the paraelectric and ferroelectric phases are under consideration, and they are built from the perfect paraelectrc and ferroelectric configurations to remove the surface Ba atoms. Results show that their magnetic moments are 0.12*µ*_B_ (0.03*μ_B_/unit-cell*) and 0.48*µ*_B_ (0.13*μ_B_/unit-cell*) respectively, and the magnetic moment in ferroelectric film is four times higher than that of paraelectric configuration, quite different from the cases of *m* = 1 and 2 that the corresponding magnetic moments of paraelectric phases are 2.00*μ_B_* (0.50*μ_B_/unit-cell*) and 1.88*μ_B_* (0.24*μ_B_/unit-cell*) respectively, indicating that the spontaneous polarization has a large influence in the magnetization.

To study the spontaneous polarization pattern of the BaTiO_3_ ultrathin film, both the density function perturbation theory (DFPT) and berry-phase approach have been employed. For DFPT, the spontaneous polarization is assumed to be mapped onto the center Ti atom of a unit cell, thereby, the spontaneous polarization of bulk BaTiO_3_ can be calculated using Equation (4) [40]:

(4) $$P_{k}=\frac{e}{\Omega_{k}}[({u_{k}}^{Ti}-u_{k0})\cdot Z^{*}{}_{Ti}+\frac{1}{2}\sum_{i=1}^{6}({u_{ki}}^{O}-u_{k0})\cdot Z^{*}{}_{O}+\frac{1}{8}\sum_{j=1}^{8}({u_{kj}}^{Ba}-u_{k0})\cdot Z^{*}{}_{Ba}],$$

where *k* denotes the Ti-centered unit cell, *u_k0_* indicates the central position of the cell, *k_i_* and *k_j_* denote the oxygen and barium atoms, *u* is the atomic position, Ω_k_ is volume, and Z* is the Born effective charge tensor.

The Born effective Charges of the bulk in different directions are listed in Table 2, which shows that the changes of the effective Born Charges of Ti and O atoms in the direction [001] are more than 1*e* relative to the cubic structure, and the charge transfer between Ti and O atoms occurs. As a result, the spontaneous polarization is calculated to be 28.29 *µ*C/cm^2^. By the berry-phase method, the cubic structure is adopted as a reference. Due to the atomic displacements, the total dipole moments of the ferroelectric phase is evaluated to be 1.14 electron·Å, thus, the spontaneous polarization is calculated to be 28.83 *µ*C/cm^2^, it is in good agreement with that gained by the DFPT method and the experiment value 26 *µ*C/cm^2^ [43]. In the following part of the paper, the DFPT method is adopted to study the ferroelectric property of the BaTiO_3_ (001) ultrathin film to evaluate the spontaneous polarization in the unit cell. 

**Figure 7:**
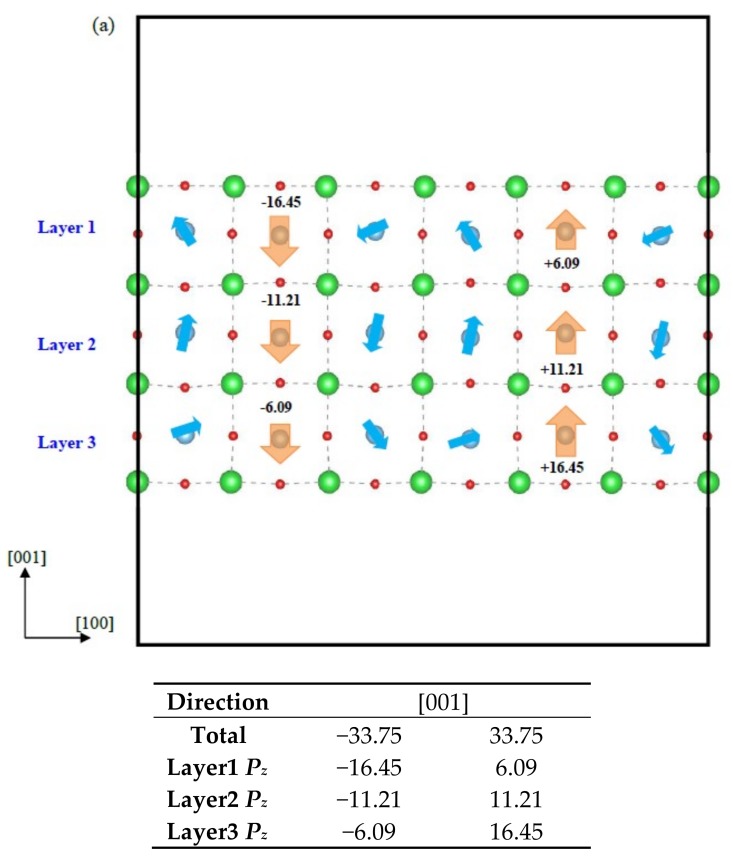

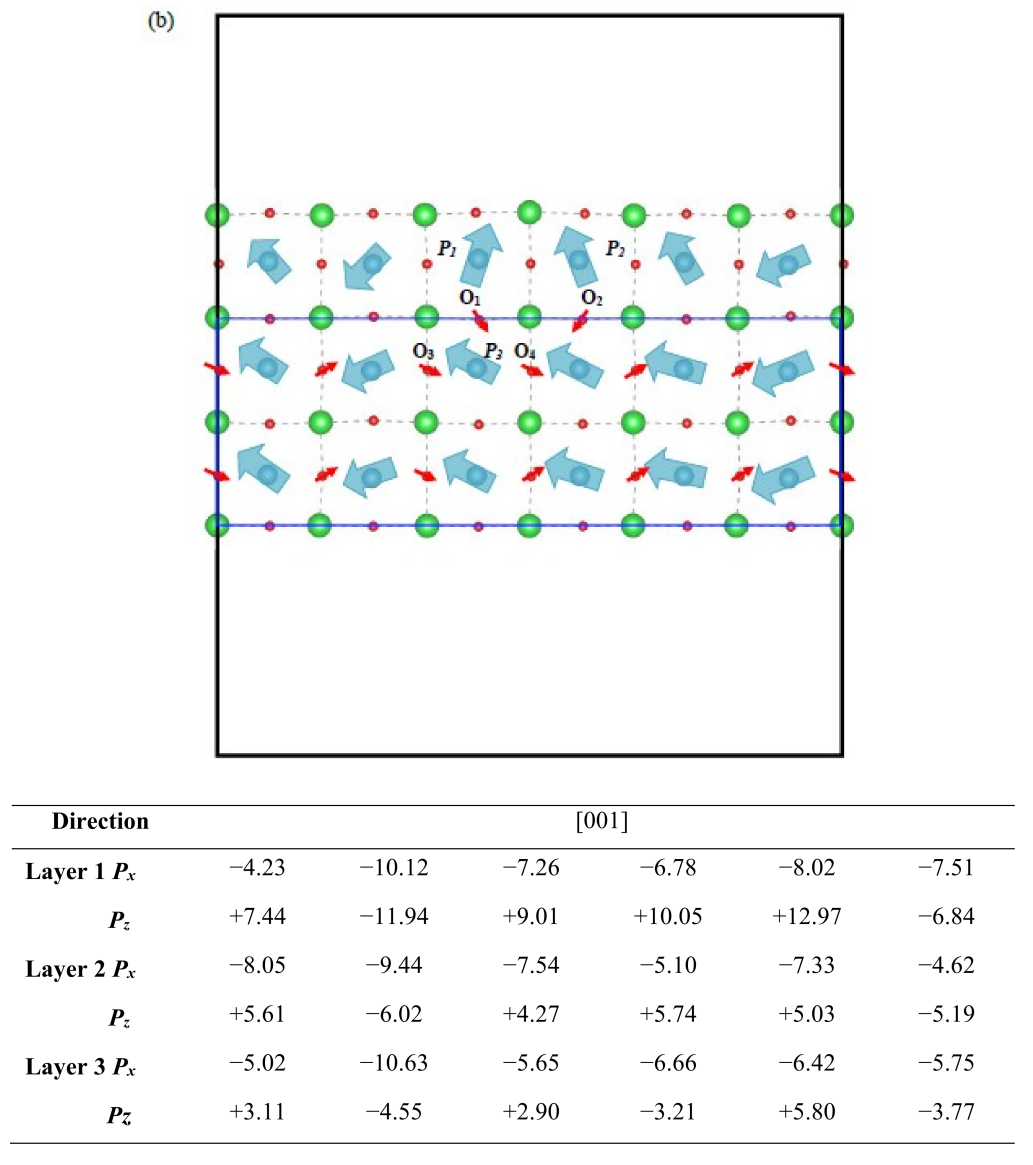

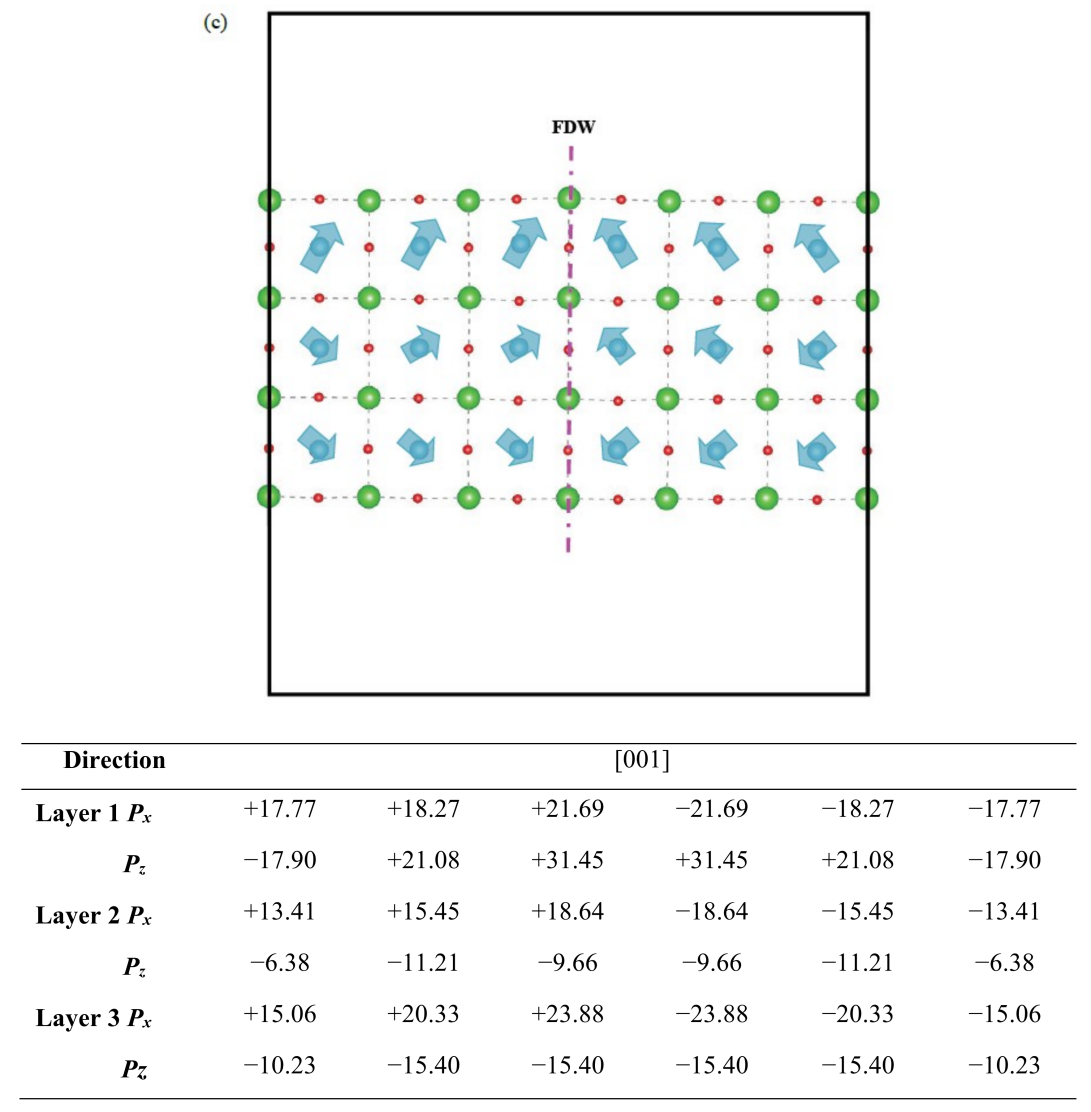
Spontaneous polarization pattern of BaTiO_3_ (001) ultrathin film with thickness *m* = 3, (**a**) perfect ultrathin film with ferroelectric phase, (**b**) Ba-deficient ultrathin film with ferroelectric phase, where the red arrow denotes the moving direction of the O atom (**c**) Ba-deficient ultrathin film with paraelectric phase. The magnitude of *P* in per unit cell is expressed in *µ*C/cm^2^.

Figure 7. is the spontaneous polarization pattern of (001) ultrathin film with thickness *m* = 3, Figure 7a is the perfect configuration with ferroelectric structure, Figure 7b,c are the Ba-deficient ones with the ferroelectric and paraelectric phases. Before giving an insight into the spontaneous polarization, the surface rumpling is studied, which reflects the polarization pattern and is defined as follows [42]:

(5) $$r=\frac{z_{O}-z_{aver}}{z_{aver}},$$

where *r* represents the surface rumpling, *z_O_* is the *z* coordination of the O atom at the surface, and *z_aver_* is the average value of the *z* coordination of the surface Ba atoms. Results show that the top surface rumpling *r* is 0.51%, 0.44%, 0.51%, 0.42%, 0.51% and 0.42% respectively from the left to the right side in the [100] direction, while the bottom surface rumpling is in a centrosymmetry with the top rumpling. This mainly results from the relaxation of surface atoms contacting vacuum that is out of the restriction by the neighboring atom. 

In the perfect configuration, a closure domain is observed, as the consequence of the minimized electrostatic energy, as shown in Figure 7a. This phenomenon is also observed in the ultrathin PbTiO_3_ film, where the *depolarizing* field is sufficiently screened by the formation of spontaneous polarization parallel to the surface, as reported by Shimada et al. [40]. Besides, a spontaneous polarization perpendicular to the surface is found, which is denoted as *P_z_*. They are calculated to be 6.09 *µ*C/cm^2^, 11.21 *µ*C/cm^2^ and 16.45 *µ*C/cm^2^ in the direction of the dipoles, respectively. Clearly, in the optimized structure, the spontaneous polarization of *P_z_* is enhanced because the surface O atom moves out of plane respective to the paraelectric phase, and the distance between Ti and O atoms is thus increased, contributing to the enhancement of spontaneous polarization. Figure 7b is the Ba-deficient configuration of ferroelectric phase, where the spontaneous polarization pattern is changed and the spontaneous polarization *P_z_* is deviated from the direction [001], as a result, the *P_x_* parallel to the surface is formed. Interestingly, all the *P_x_* in the blue box are orientated to the same direction, as a result, the closure domain and ferroelectric domain wall (FDW) disappear. To interpret this phenomenon, the corresponding optimized geometric structure is analyzed. When the surface Ba vacancy is produced, the surrounded O atoms will move away from the vacancy site, resulting in the formation of the *P*_1_ and *P*_2_, and they seem to be symmetric. Meanwhile, this causes the O_1_ and O_2_ atoms to move towards each other or the Ba atom in the direction [100] as well. In the direction [001], they move downwards, as shown in Figure 7b, leading to the spontaneous polarization *P*_3_ to realign. Consequently, the O_3_ and O_4_ constituting *P*_3_ move rightwards in the direction [100], which induces the Ti atom in the neighboring unit cell to move towards the opposite direction of the movement of O_4_ because of the ionic polarization between the Ti and O_4_, and makes the FDW disappear. Here, the paraelectric phase is adopted as a reference to confirm the coupling between the magnetic moment and spontaneous polarization. Its spontaneous polarization pattern is also studied, as shown in Figure 7c. Different from the ferroelectric film, an FDW is also observed in this film. It can be illustrated that when the surface Ba atom is removed from the perfect paraelectric film, the movement of atoms shows mirror symmetry along the FDW, which is also the result of minimizing the electrostatic energy. From Figure 7b,c, it can be seen clearly that the magnitude of spontaneous polarization *P_x_* and *P_z_* in per cell in the ferroelectric film are lower than that in paraelectric configuration. For example, the *P_z_* are calculated to be 7.44 *µ*C/cm^2^, −11.94 *µ*C/cm^2^, 9.01 *µ*C/cm^2^, 10.05 *µ*C/cm^2^, 12.97 *µ*C/cm^2^ and −6.84 *µ*C/cm^2^ respectively from the left to the right along the direction [100] in the first layer, while that are −17.90 *µ*C/cm^2^, 21.08 *µ*C/cm^2^, 31.45 *µ*C/cm^2^, 31.45 *µ*C/cm^2^, 21.08 *µ*C/cm^2^ and −17.90 *µ*C/cm^2^ in the paraelectric film. Generally, the corresponding *P_x_* is also lower compared with the paraelectric phase. In addition, the Ba-deficient ferroelectric film is more energetically stable relative to the paraelectric one confirmed from the total energies, meaning that the spontaneous polarization pattern of *P_x_* orientated to the same direction can decrease the total energy owing to the disappearance of FDWs. In order to investigate the interaction between the ferroelectric and ferromagnetic orders which are both involved by the O atoms, the evaluations of magnetic moments as a function of spontaneous polarization are carried out using the spin-orbit-coupling (SOC) method. For thickness *m* = 3, the partial magnetic moments in the ferroelectric film are 0.28*µ*_B_ and 0.31*µ*_B_ respectively in the direction [100] and [001], while that is only 0.06*µ*_B_ and 0.07*µ*_B_ in the paraelectric configuration. It is concluded that the magnetic moment is enhanced when the spontaneous polarization is decreased. To further confirm it, the spontaneous polarization and magnetic moment as a function of stress are studied, that’s because the spontaneous polarization is sensitive to the external stress. Here bi-axial stress is applied to the ultrathin film with *m* = 2, and Table 3 is the magnetic moment as a function of stress. It shows that when the compress stress is applied, the magnetic moment is decreased, and when the strain achieves to −2.0%, the magnetic moment has its minimum value 1.62*µ*_B_. With increasing the stress unceasingly, the magnetic moment is found to be enhanced. Interestingly, the SOC calculation shows that in the direction [001] the magnetic moment is decreased from 1.42*µ*_B_ to 0.80*µ*_B_, and the corresponding spontaneous polarization is enhanced from 12.84 *µ*C/cm^2^ to 23.99 *µ*C/cm^2^, while in the direction [100] the magnetic moment is increased from 1.22*µ*_B_ to 1.51*µ*_B_, and the spontaneous polarization is decreased from 9.31 *µ*C/cm^2^ to 3.41 *µ*C/cm^2^ under the compress stress, as listed in Table 4. These results reveal that a conversion between the magnetic moment and spontaneous polarization occurs, meaning that the Ba-deficient BaTiO_3_ behaves in the manner of multiferroic materials. Besides that, we also do an estimation of the conversion efficiency between the spontaneous polarization and magnetic moment, which is defined as follows:

(6) $$\eta =\frac{\Delta M}{M}/\frac{\Delta P}{P},$$

where *η* is the conversion efficiency, *P* and *M* is the partial spontaneous polarization and magnetic moment.

Clearly, the partial conversion efficiency in the direction [100] and [001] is calculated to be 35.22%, 38.57% and 37.51%, and 63.56%, 74.21% and 50.28% respectively, corresponding to the strain of −1%, −2% and −3%, indicating that a strong ME coupling occurs in the deficient BaTiO_3_ ultrathin film with thickness *m* = 2. In the future, the influence of external stress field on the multiferroic property will be studied in detail. Summarily, the BaTiO_3_ (001) ultrathin film with surface Ba vacancy behaves in the manner of multiferroic materials, and the corresponding ME coupling is strong. It may stimulate more interesting study to explore new multiferroic material systems based on the perovskite structure, which would resolve the weak magnetoelectric coupling in the BiFeO_3_.

## 4. Conclusions 

In this work, we have performed a first-principles study on ME coupling in two-dimensional BaTiO_3_ (001) ultrathin film with surface Ba vacancy. Results show that the (101) configuration is the most energetically stable, but the electronic density of states suggests that it behaves like a conductor. Although both the (111) and (001) films are semiconductors, the (001) configuration has a lower formation energy, indicating that it is more energetically stable. Owing to the slight difference between the surface grand potentials of the (001) ultrathin films with the BaO- and TiO_2_- terminations, the configuration with BaO- termination is adopted to study the ME coupling. 

Taking surface vacancy into account, band structures corresponding to the O and Ba vacancies have been calculated, which show that the O vacancy can switch the ultrathin film into a metal, while the Ba vacancy has a slight influence on the Sham-Kohn band gap, at a ～2.13% defect concentration. In the Ba-deficient configuration, the spontaneous polarization pattern is changed and a spontaneous polarization parallel to the surface is observed. In the meantime, a magnetic moment is induced, and it is confirmed to be originated from the O atoms. The spin charge distribution and SOC calculation demonstrate that the coupling between the ferroelectric and ferromagnetic orders has a major influence in the magnetization. It is confirmed that the partial conversion efficiency of spontaneous polarization converting into magnetic moment in the direction [100] and [001] are more than 35.22% under the bi-axial compress stress, revealing that a strong ME coupling occurs in the deficient BaTiO_3_ ultrathin film with thickness *m* = 2. That’s to say, it behaves in the manner of good multiferroic performance.

## Figures and Tables

**Table 1:** Surface grand potentials of BaTiO_3_ ultrathin films with different terminations (*J/m*^2^).

(001)		(101)		(111)	
BaO-	TiO_2_-	BaTiO-	O_2_-	BaO_3_-	Ti-
−55.51	−56.20	−75.52	−62.12	−41.63	−38.38

**Table 2:** Effective Born charges calculated by DFPT method of the tetragonal and cubic BaTiO_3_ structures.

Atom	Tetragonal			Cubic	
	Z_xx_^*^	Z_yy_^*^	Z_zz_^*^	Z^*^	
O_I_	−2.14	−5.70	−2.01	−2.15(O_⊥_)	−5.87(O_‖_)
O_II_	−2.01	−2.01	−4.63	−2.15(O_⊥_)	−5.87(O_‖_)
Ti	7.10	7.10	5.67	7.42	
Ba	2.73	2.73	2.84	2.75	

**Table 3:** Magnetic moment as a function of the bi-axial compress strain in the ultrathin Ba-deficient BaTiO_3_ film with *m* = 2.

Strain (%)	Magnetic Moment (*μ_B_*)
0	1.81
−1	1.73
−2	1.62
−3	1.71

**Table 4:** Partial magnetic moment and spontaneous polarization in the directions [100] and [001] in the ultrathin Ba-deficient BaTiO_3_ film with *m* = 2 under compress stress.

Strain (%)	Magnetic Moment (*μ_B_*)		Spontaneous Polarization (*µ*C/cm^2^)	
	Direction [100]	Direction [001]	Direction [100]	Direction [001]
0	1.22	1.42	9.31	12.84
−1	1.25	1.20	8.66	15.97
−2	1.36	0.88	6.54	19.42
−3	1.51	0.80	3.41	23.99

## Supplementary Materials

The following are available online at http://www.mdpi.com/2079-4991/9/2/269/s1, Figure S1: The BaTiO_3_ (101) ultrathin films with paraelectric structures and (a) BaTiO- (b) O_2_- terminations, Figure S2: The BaTiO_3_ (111) ultrathin films with paraelectric structures and (a) BaO_3_- (b) Ti- terminations.
