# Supplementary material for: A First-Principles Study on the Multiferroic Property of Two-Dimensional BaTiO3 (001) Ultrathin Film with Surface Ba Vacancy
Source: Nanomaterials (Basel). 2019 Feb 15;9(2):269. doi: 10.3390/nano9020269 (PMC6410265; doi:10.3390/nano9020269)
Supplement: Supplementary file 1 [file nanomaterials-09-00269-s001.pdf]

Supplementary materials

The simulation model of $BaTiO_3$ (101) and (111) ultrathin films with dimension of 1×1×6

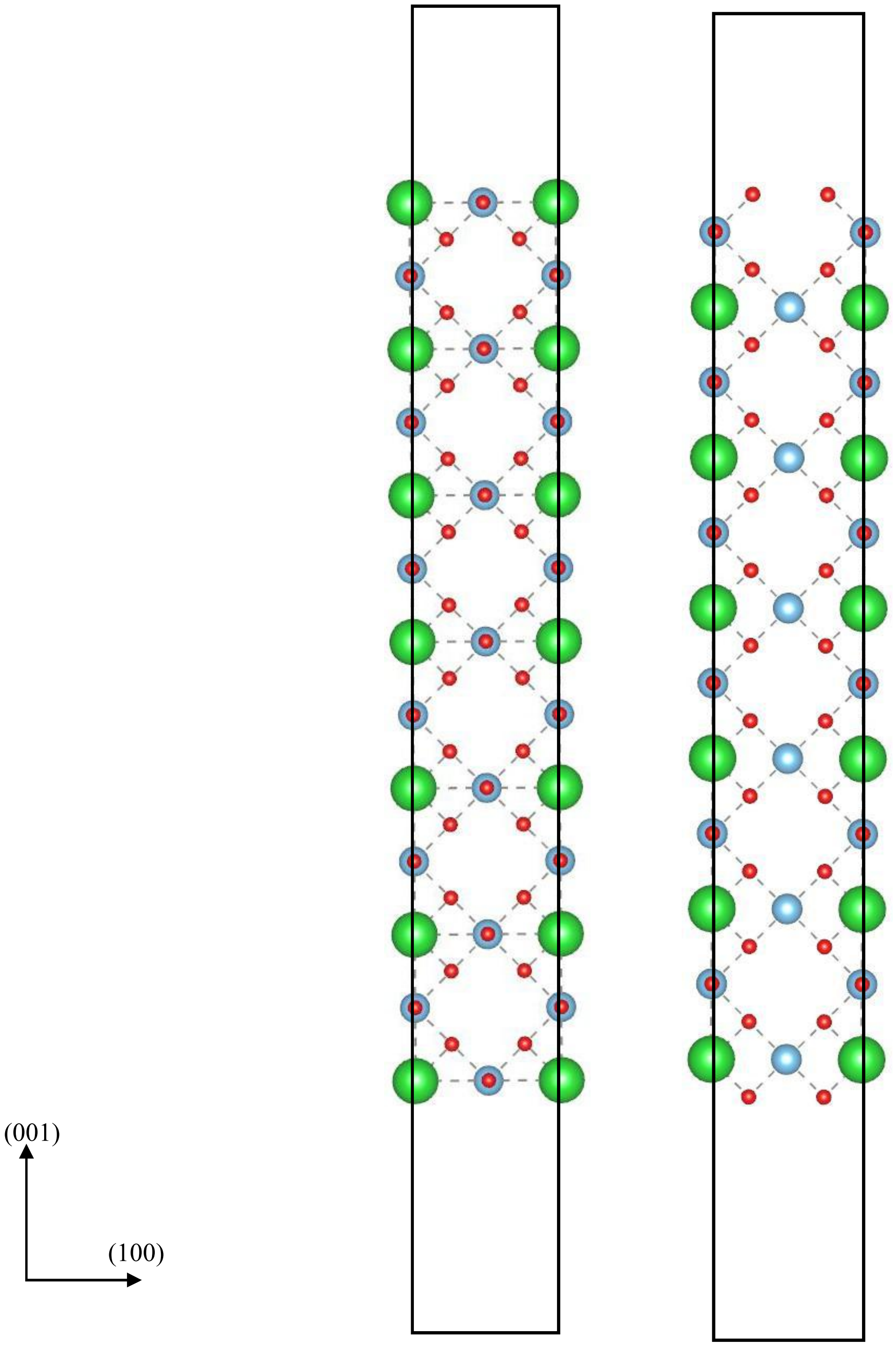

Figure 1 The $BaTiO_3$ (101) ultrathin films with paraelectric structures and
(a) BaTiO- (b) O_2- terminations

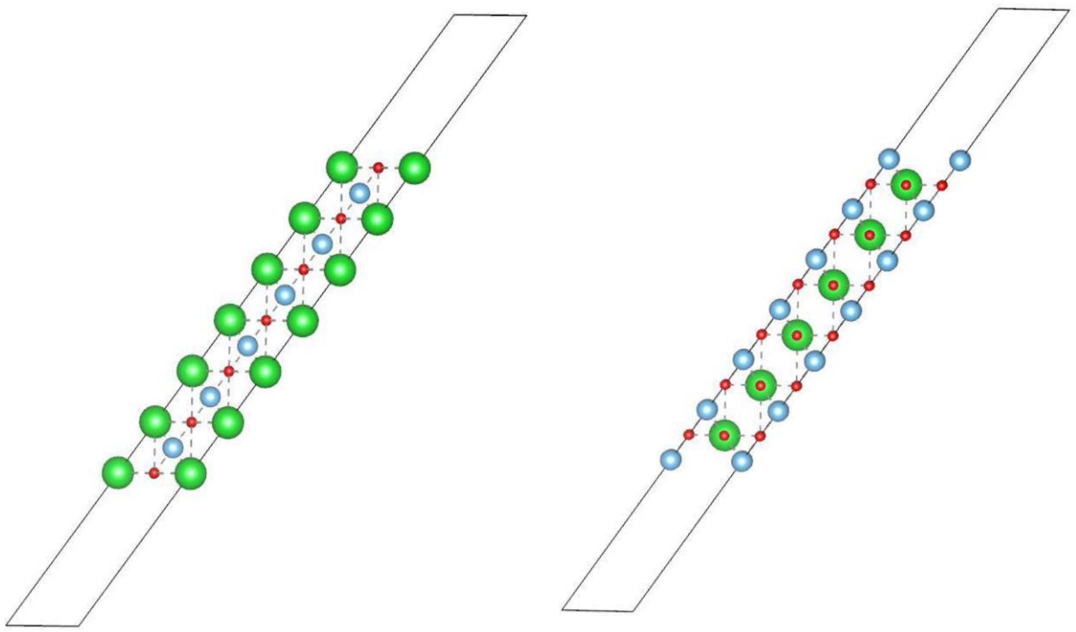

Figure 2 The $BaTiO_3$ (111) ultrathin films with paraelectric structures and (a) BaO_3- (b) Ti- terminations
